# Supplementary material for: Extensive regulation of enzyme activity by phosphorylation in Escherichia coli
Source: Nat Commun. 2021 Sep 24;12:5650. doi: 10.1038/s41467-021-25988-4 (PMC8463566; doi:10.1038/s41467-021-25988-4)
Supplement: Supplementary file 1 — Supplementary Information File [file 41467_2021_25988_MOESM1_ESM.pdf]

Supplementary Information for

“Extensive regulation of enzyme activity by phosphorylation in *Escherichia coli*”.

Schastnaya et al.

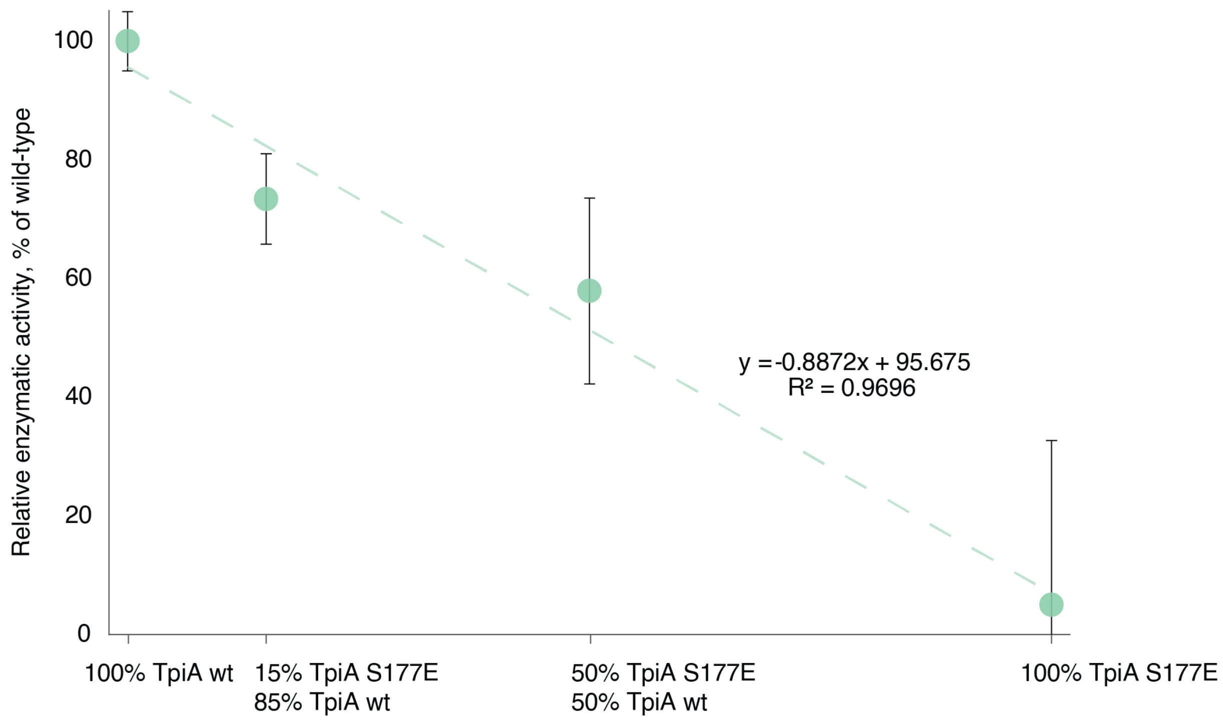

Supplementary Figure 1 | The influence of mimicking TpiA S177 phosphosite occupancy on enzymatic activity. Enzymatic activities of four samples with different ratios between the unphosphorylated wild-type (TpiA wt) and S177E phosphomimicking mutant (TpiA S177E), approximating 0, 15, 50, and 100% occupancy of S177 phosphorylation site. The activity values were normalized to the sample containing 100% TpiA wild-type. The substrate concentration was 1 mM dihydroxyacetone phosphate. Data represent the mean ± standard deviation. The number of independent replicates over two experiments was six for 100% TpiA wt, seven for mixed samples, and four for 100% TpiA S177E. Source data are provided as a Source Data file.

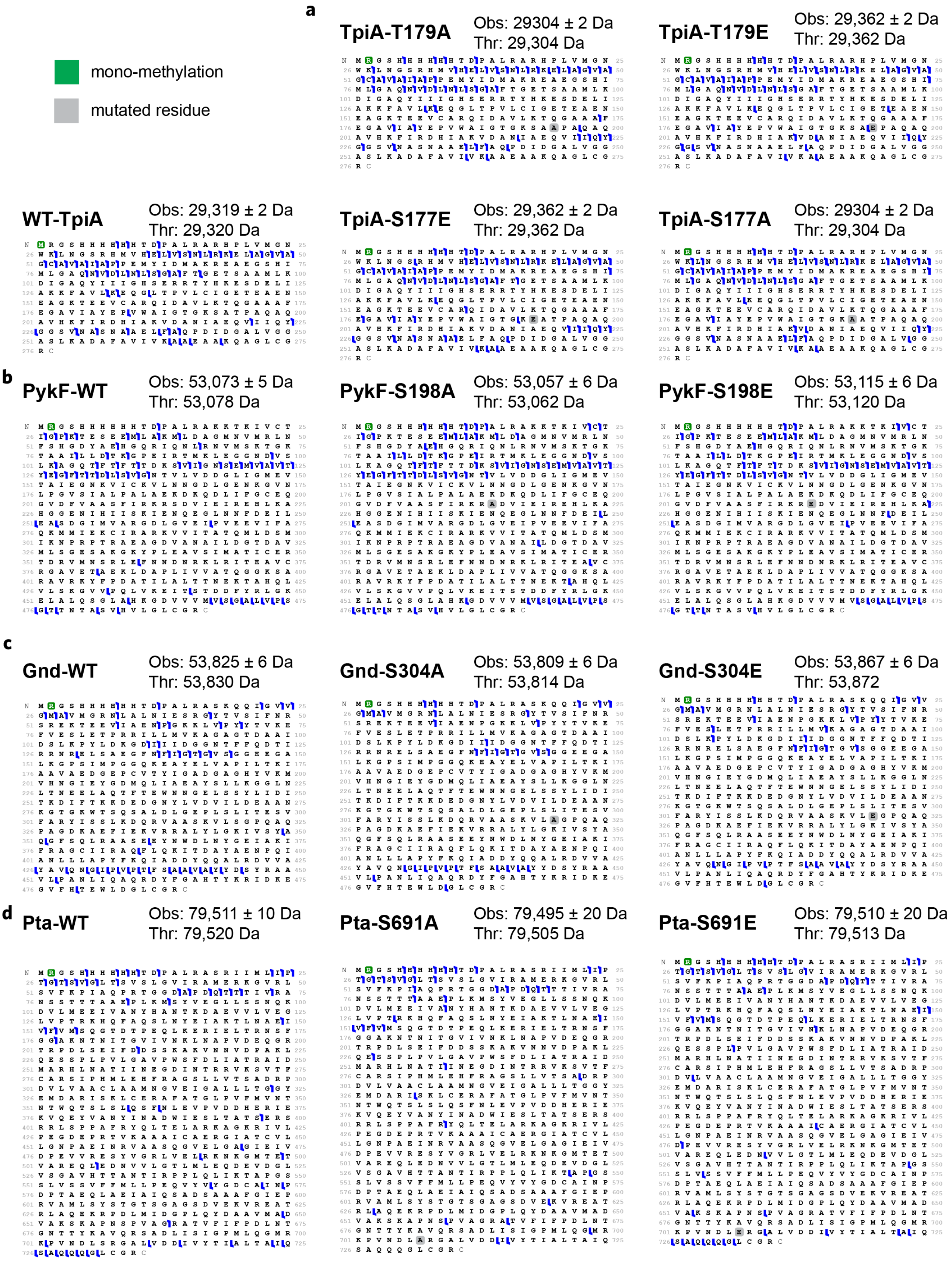

Supplementary Figure 2 | PTM screening of wild-type and mutant intact TpiA, PykF, Gnd and Pta. Fragmentation maps for wild-type (wt) and mutated **a**, TpiA, **b**, PykF, **c**, Gnd and **d**, Pta are shown with observed (Obs) and theoretical (Thr) masses. Experimental standard deviations for the intact protein masses are provided.

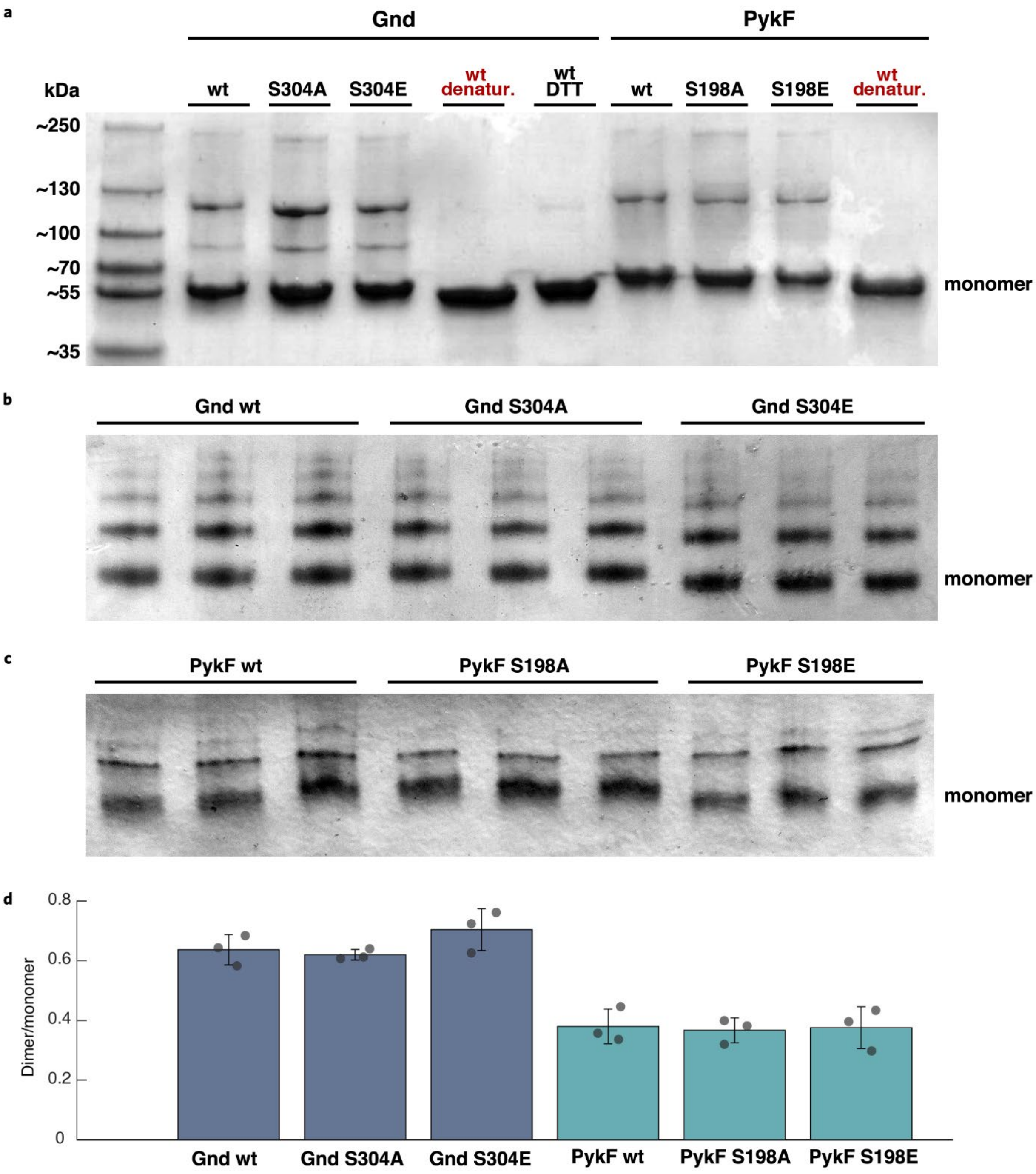

Supplementary Figure 3 | Mono/multimer composition of purified overexpressed wild-type and phosphomutant enzymes. **a**, SDS-PAGE of Gnd and PykF wild-type (wt) and phosphomutants in native (black) and denaturing (red) sample buffer. Samples in denaturing buffer were heated to 95 °C for 10 min. Native Gnd wt sample was additionally treated with DTT without heat treatment (wt DTT) to break down the oligomers. Similar results were obtained in two independent experiments. **b**,**c**, Native PAGE of Gnd and PykF wt and phosphomutants in native sample buffer. The purity and composition of the samples was previously confirmed by SDS-PAGE (**a**), samples were run in triplicates. **d**, Dimer/monomer ratio in native conditions estimated via densitometry. Data represent the mean ± standard deviation, and individual replicates (3) are shown. Source data are provided as a Source Data file.
