# Supplementary material for: Extensive regulation of enzyme activity by phosphorylation in Escherichia coli
Source: Nat Commun. 2021 Sep 24;12:5650. doi: 10.1038/s41467-021-25988-4 (PMC8463566; doi:10.1038/s41467-021-25988-4)
Supplement: Supplementary file 3 — Description of Additional Supplementary Information [file 41467_2021_25988_MOESM3_ESM.pdf]

Description of Additional Supplementary Information File

File name: Supplementary data 1

Description: Phosphomutant strains

File name: Supplementary data 2

Description: Growth rates of phosphomutant and knockout strains

File name: Supplementary data 3

Description: Changes in metabolites for phosphomutant and knockout strains

File name: Supplementary data 4

Description: Local metabolic changes

File name: Supplementary data 5

Description: Spearman correlation of metabolic profiles

File name: Supplementary data 6

Description: Melting temperatures of purified enzymes

File name: Supplementary data 7

Description: *In vitro* activity of phosphomutants enzymes

File name: Supplementary data 8

Description: Oligonucleotides used to create genomic phosphomutants and mutant ASKA plasmids
