# Supplementary material for: Extensive regulation of enzyme activity by phosphorylation in Escherichia coli
Source: Nat Commun. 2021 Sep 24;12:5650. doi: 10.1038/s41467-021-25988-4 (PMC8463566; doi:10.1038/s41467-021-25988-4)
Supplement: Supplementary file 12 — Reporting Summary [file 41467_2021_25988_MOESM12_ESM.pdf]

nature research

Corresponding author(s): Prof. Dr. Uwe Sauer

Last updated by author(s): August 25, 2021

Reporting Summary

Nature Research wishes to improve the reproducibility of the work that we publish. This form provides structure for consistency and transparency in reporting. For further information on Nature Research policies, see our Editorial Policies and the Editorial Policy Checklist.

Statistics

For all statistical analyses, confirm that the following items are present in the figure legend, table legend, main text, or Methods section.

n/a	Confirmed	
☐	☒	The exact sample size (*n*) for each experimental group/condition, given as a discrete number and unit of measurement
☐	☒	A statement on whether measurements were taken from distinct samples or whether the same sample was measured repeatedly
☐	☒	The statistical test(s) used AND whether they are one- or two-sided<br>*Only common tests should be described solely by name; describe more complex techniques in the Methods section.*
☒	☐	A description of all covariates tested
☐	☒	A description of any assumptions or corrections, such as tests of normality and adjustment for multiple comparisons
☐	☒	A full description of the statistical parameters including central tendency (e.g. means) or other basic estimates (e.g. regression coefficient) AND variation (e.g. standard deviation) or associated estimates of uncertainty (e.g. confidence intervals)
☐	☒	For null hypothesis testing, the test statistic (e.g. *F*, *t*, *r*) with confidence intervals, effect sizes, degrees of freedom and *P* value noted<br>*Give P values as exact values whenever suitable.*
☒	☐	For Bayesian analysis, information on the choice of priors and Markov chain Monte Carlo settings
☒	☐	For hierarchical and complex designs, identification of the appropriate level for tests and full reporting of outcomes
☐	☒	Estimates of effect sizes (e.g. Cohen's *d*, Pearson's *r*), indicating how they were calculated

Our web collection on statistics for biologists contains articles on many of the points above.

Software and code

Policy information about availability of computer code

Data collection	Growth profiles and enzyme activity assays were recorded using Tecan i-control 3.9.1.0. Thermal denaturation of proteins was recorded using QuantStudio Design & Analysis Software v1.4.3.
Data analysis	Mass spectrometry metabolomics data processing, statistical analysis and calculation of Spearman correlation coefficients were performed in Matlab R2020b. Metabolite locality analysis was performed using MetaboSignal R Package in RStudio 1.2.5033. Densitometry analysis was performed in ImageJ 1.50e. Mass spectrometry proteomics data was analysis with UniDec 4.4.1, ProSight Lite 1.4, and FreeStyle 1.7 (Thermo Fisher). Flux analysis was performed in FiatFlux 1.66.

For manuscripts utilizing custom algorithms or software that are central to the research but not yet described in published literature, software must be made available to editors and reviewers. We strongly encourage code deposition in a community repository (e.g. GitHub). See the Nature Research guidelines for submitting code & software for further information.

Data

Policy information about availability of data

All manuscripts must include a data availability statement. This statement should provide the following information, where applicable:

- Accession codes, unique identifiers, or web links for publicly available datasets
- A list of figures that have associated raw data
- A description of any restrictions on data availability

Data are available in the Supplementary Tables and Figures. The mass spectrometry proteomics data have been deposited to the ProteomeXchange Consortium via the PRIDE partner repository with the dataset identifier PXD027243. The metabolomics and flux analysis data are available via MassIVE with the identifier MSV000087795. Source data are provided with this paper.

Field-specific reporting

Please select the one below that is the best fit for your research. If you are not sure, read the appropriate sections before making your selection.

- [x] Life sciences
- [ ] Behavioural & social sciences
- [ ] Ecological, evolutionary & environmental sciences

For a reference copy of the document with all sections, see nature.com/documents/nr-reporting-summary-flat.pdf

Life sciences study design

All studies must disclose on these points even when the disclosure is negative.

Sample size	Sample sizes were not statistically predetermined and were chosen based on the standards in the field for producing results with sufficient accuracy. All experiments were performed at least in triplicates, and the sample sizes were increased whenever possible.
Data exclusions	No data were excluded from the analyses.
Replication	Biological replicates were performed independently at least three times. All replication attempts were successful. Growth experiments were additionally replicated on separate days with fresh media. No replicates were discarded from analyses.
Randomization	Randomization was not relevant for microbiological and in vitro assays as the measurements were automated and the results were independent of the reading sequence. For metabolomics mass spectrometry measurements, the samples were injected in a randomized order.
Blinding	The investigators were not blinded during data acquisition for practical reasons. Data analysis procedures were automated and equal for all samples.

Reporting for specific materials, systems and methods

We require information from authors about some types of materials, experimental systems and methods used in many studies. Here, indicate whether each material, system or method listed is relevant to your study. If you are not sure if a list item applies to your research, read the appropriate section before selecting a response.

Materials & experimental systems

n/a	Involved in the study	
x		Antibodies
x		Eukaryotic cell lines
x		Palaeontology and archaeology
x		Animals and other organisms
x		Human research participants
x		Clinical data
x		Dual use research of concern

Methods

n/a	Involved in the study	
x		ChIP-seq
x		Flow cytometry
x		MRI-based neuroimaging
